# Benchmarking palliative care practices in neurooncology: a german perspective

**DOI:** 10.1007/s11060-024-04674-7

**Published:** 2024-05-02

**Authors:** Anna Cecilia Lawson McLean, Aaron Lawson McLean, Thomas Ernst, Marie-Therese Forster, Christian Freyschlag, Jens Gempt, Roland Goldbrunner, Stefan Grau, Christine Jungk, Birgitt van Oorschot, Steffen K. Rosahl, Ulrich Wedding, Christian Senft, Marcel A. Kamp, on behalf of the German Consortium for Excellence in Neurooncology, Palliative Care (GCE-NPC), Martin Misch, Ulrich Herrlinger, Vesna Malinova, Marcia Machein, Peter Hau, Oliver Grauer, Martin Glas, Almuth F. Kessler, Naureen Keric, Hannes Egermann, Marco Stein, Jens Weigel, Marcus Reinges, Andreas Jödicke, Klaus-Peter Stein, Marcus Mehlitz, Sven-Axel May, Niklas Thon, Rebecca Kassubek, Ulrich Knappe, Ali Alomari, Florian H. Ebner, Mirjam Renovanz, Elisabeth Bumes, Clemens Seidel, Hans Clusmann, Thomas M. Freiman, Yu-Mi Ryang, Julia Gerhardt, Michael Stoffel, Ina Lange, Volker Tronnier, Walter Schulz-Schaeffer

**Affiliations:** 1 Department of Neurosurgery, Jena University Hospital, Friedrich Schiller University Jena, Jena, Germany; 2 University Tumor Center (UTC), Jena University Hospital, Jena, Germany; 3 Department of Neurosurgery, University Hospital Frankfurt, Frankfurt, Germany; 4 Department of Neurosurgery, Medical University of Innsbruck, Innsbruck, Austria; 5 Department of Neurosurgery, University Hospital Hamburg Eppendorf, Hamburg, Germany; 6 Center for Neurosurgery, University Hospital Cologne, Cologne, Germany; 7 Department of Neurosurgery, Klinikum Fulda, Fulda, Germany; 8 Department of Neurosurgery, Medical Faculty, Heidelberg University, Heidelberg, Germany; 9 Department of Palliative Care, University Hospital Wurzburg, Wurzburg, Germany; 10 Department of Neurosurgery, Helios Klinikum and Health Medical University Erfurt, Erfurt, Germany; 11 Department of Palliative Care, Jena University Hospital, Jena, Germany; 12 Comprehensive Cancer Center Central Germany (CCCG), Jena/Leipzig, Germany; 13 Department of Palliative Care and Neuro-Palliative Care, Brandenburg Medical School Theodor Fontane, Immanuel Klinik Rüdersdorf, Neuruppin, Germany; 14 Department of Neurosurgery, Charité, University Hospital Berlin, Berlin, Germany; 15 Division of Clinical Neurooncology, Department of Neurology and Center of Integrated Oncology (CIO), Bonn, Germany; 16 Department of Neurosurgery, University Hospital Göttingen, Göttingen, Germany; 17 Department of Neurosurgery, University Hospital Freiburg, Freiburg, Germany; 18 Department of Neurology, University Hospital Regensburg, Regensburg, Germany; 19 Department of Neurology, University Hospital Münster, Münster, Germany; 20 Department of Clinical Neurooncology, Department of Neurology, University Hospital Essen, Essen, Germany; 21 Department of Neurosurgery, University Hospital Würzburg, Würzburg, Germany; 22 Department of Neurosurgery, University Hospital Mainz, Mainz, Germany; 23 Department of Neurosurgery, Barmherzige Brüder Hospital, Regensburg, Germany; 24 Neurooncological Centre, University Hospital Gießen/Marburg, Campus Gießen, Gießen, Germany; 25 Department of Neurosurgery, Paracelsus Private Medical School, Nuremburg, Germany; 26 Department of Neurosurgery, Hospital Bremen-Mitte, Bremen, Germany; 27 Neurooncological Center, CCBN, Department of Neurosurgery, Vivantes Hosiptal, Berlin, Germany; 28 Department of Neurosurgery, University Hospital Magdeburg, Magdeburg, Germany; 29 Department of Neurosurgery, Barmherzige Brüder Hospital, Trier, Germany; 30 Department of Neurosurgery, Chemnitz Hospital, Chemnitz, Germany; 31 Department of Neurosurgery, University Hospital of the Lukas-Maximilian-UniversityMunich, Munich, Germany; 32 Department of Neurology, University Hospital Ulm, Ulm, Germany; 33 Neurooncological Center MiNOZ, Johannes Wesling Medical Center Minden, UK RUB, Minden, Germany; 34 Department of Neurosurgery, Alfried Krupp Hospital, Essen Rüttenscheid, Essen, Germany; 35 Neurooncological Center, University Hospital Tübingen, Tübingen, Germany; 36 Elisabeth Bumes, Wilhelm Sander-NeuroOncology Unit, Regensburg University Hospital, Regensburg, Germany; 37 Department of Radiooncology, University Hospital Leipzig, Leipzig, Germany; 38 Department of Neurosurgery, University Hospital RWTH Aachen, Aachen, Germany; 39 Department of Neurosurgery, University Hospital Rostock, Rostock, Germany; 40 Department of Neurosurgery, Helios Hospital Berlin Buch, Berlin, Germany; 41 Department of Neurosurgery, Helios Hospital Krefeld, Krefeld, Germany; 42 Neurooncological Center Greifswald, University Hospital Greifswald, Greifswald, Germany; 43 Department of Neurosurgery, University Hospital Schleswig-Holstein, Campus Lübeck, Lübeck, Germany; 44 Institute of Neuropathology, University Hospital Homburg/Saar, Homburg, Germany

**Keywords:** Palliative care, Neurooncology, Quality of life

## Abstract

**Purpose:**

To benchmark palliative care practices in neurooncology centers across Germany, evaluating the variability in palliative care integration, timing, and involvement in tumor board discussions. This study aims to identify gaps in care and contribute to the discourse on optimal palliative care strategies.

**Methods:**

A survey targeting both German Cancer Society-certified and non-certified university neurooncology centers was conducted to explore palliative care frameworks and practices for neurooncological patients. The survey included questions on palliative care department availability, involvement in tumor boards, timing of palliative care integration, and use of standardized screening tools for assessing palliative burden and psycho-oncological distress.

**Results:**

Of 57 centers contacted, 46 responded (81% response rate). Results indicate a dedicated palliative care department in 76.1% of centers, with palliative specialists participating in tumor board discussions at 34.8% of centers. Variability was noted in the initiation of palliative care, with early integration at the diagnosis stage in only 30.4% of centers. The survey highlighted a significant lack of standardized spiritual care assessments and minimal use of advanced care planning. Discrepancies were observed in the documentation and treatment of palliative care symptoms and social complaints, underscoring the need for comprehensive care approaches.

**Conclusion:**

The study highlights a diverse landscape of palliative care provision within German neurooncology centers, underscoring the need for more standardized practices and early integration of palliative care. It suggests the necessity for standardized protocols and guidelines to enhance palliative care's quality and uniformity, ultimately improving patient-centered care in neurooncology.

**Supplementary Information:**

The online version contains supplementary material available at 10.1007/s11060-024-04674-7.

## Introduction

Neurooncology is a rapidly evolving field, with advances in surgical techniques, radiation therapy, and systemic therapy resulting in improved outcomes for patients with brain tumors. Despite these encouraging strides, the management of malignant brain tumors continues to present significant challenges. The inherent high morbidity and mortality rates of these tumors, combined with the unique needs of this patient population, necessitate novel and comprehensive approaches, encompassing palliative, spiritual and psycho-oncological care [1]. For example, there is a lack of consensus regarding the optimal timing of involvement and referral to palliative care, and little is known about the availability and utilization of palliative and psycho-oncology services in centers providing neurooncology care [2–5].

Since 2008, Germany has seen significant progress in oncological care structuring and quality assurance, particularly with the development of the National Cancer Plan for achieving cross-sector, integrated oncological care [6]. Three significant parallel but interconnected structures have evolved, each established or supported by different providers:

The German Cancer Society (DKG) has established a center model and certifies these oncological centers. These certified centers form networks that include both inpatient and outpatient facilities, fostering close collaboration among all disciplines involved in cancer patient treatment. Rigorous criteria for structure, process, and outcome quality have been defined for these centers and their certification. Typically, larger centers comprise an oncology center along with specialized organ cancer centers. Organ cancer centers focus on treating cancers originating from specific organs (e.g., lung, bladder). Additionally, when a certain percentage of oncological patients are treated in certified centers, an oncological center can attain certification as an umbrella organization, facilitating the comprehensive treatment of various tumor types under one roof. The certification is available to all hospitals, whether university-affiliated or non-university institutions [7].

Key quality criteria for neurooncology centers encompass specifying case numbers, making treatment decisions through interdisciplinary tumor boards comprising neurosurgeons, neurologists, radiation therapists, oncologists, neuroradiologists, and neuropathologists, implementing evidence-based treatments following guidelines, and actively involving patients in clinical studies. Certified centers are also expected to host specialized diagnostic and therapeutic facilities, emphasizing on providing comprehensive neurooncological care (https://www.onkozert.de/organ/neuro/, date of access November 1st 2022).

The creation of certified oncology centers constitutes one of several prerequisites for Comprehensive Cancer Centers (CCC) situated at university hospitals with a focus on oncology, established by the German Cancer Aid, which is a non-profit cancer organization dedicated to funding research, supporting patient care, and promoting awareness and prevention. The establishment of these CCCs is geared towards enhancing personalized cancer treatment and spearheading the development of innovative therapeutic strategies. The third pillar, the National Centers for Tumor Diseases (NCT), supported by the Federal Ministry of Education and Research (BMBF), shares the goal of swiftly and securely translating promising results from cancer research into clinical applications and pioneering treatment approaches. Together, these initiatives underscore a comprehensive approach to oncological care, balancing cutting-edge research with patient-centered treatment methodologies [8].

An integral component of neurooncological treatment across these structures is palliative care. For a neurooncology center to obtain successful DKG certification, it must demonstrate robust palliative care infrastructures [9]. This includes the establishment of a dedicated palliative care unit, the employment of physicians and staff with specialized qualifications in palliative care, maintaining an appropriate staffing ratio, the ability to provide urgent palliative medical care within a 30-min timeframe, and the promotion of a multi-professional approach to treatment. Furthermore, key performance indicators for these centers are centered around the assessment of palliative burden, utilizing established instruments such as the Minimal Documentation System (MIDOS) or the Integrated Palliative Care Outcome Scale (IPOS), alongside psycho-oncological evaluation tools like the distress thermometer. Despite these structured requirements, several critical facets of palliative care, notably the optimal timing for its integration, comprehensive quality of life assessments, and the incorporation of advanced care planning or spiritual care, remain inadequately defined within the existing framework.

Moreover, the landscape of palliative care in neurooncology is not homogeneous across Germany and not all hospitals feature DKG-certified neurooncology centers. There is an assumption that a variety of palliative care concepts are in practice across both DKG-certified and university hospitals, yet a uniform presence of these palliative care structures is not universally evident in all such institutions. This study, therefore, aims to evaluate the structures and methodologies of neurooncological palliative medicine within both DKG-certified and university-based neurooncology centers in Germany. By benchmarking these practices, we seek to illuminate the current landscape of palliative care in neurooncology, identify gaps and variances in care delivery, and contribute to the broader discourse on optimal palliative care practices in neurooncology.

## Methods

### Ethics approval and data availability

This study strictly adhered to the ethical principles outlined in the 1964 Helsinki Declaration and its subsequent amendments. The study was approved by the ethics committee of the Jena University Hospital (reference number: 2022–2753-Bef) and all respondents provided informed consent. Anonymous survey responses were permitted to protect the privacy of the participants. The anonymized data are available upon reasonable request.

### Study design

We conducted a national survey at both DKG-certified and non-certified academic neurooncology centers with the goal of identifying the structures and concepts of palliative care for patients with neurooncological conditions.

### Survey development and distribution

The survey was developed and hosted on an internet platform (Nextcloud Version 24.0.4 Enterprise, Nextcloud GmbH, Stuttgart, Germany). An English translation of the survey is provided (Supplementary file 2).

The survey link was distributed to the head and / or coordinator of all DKG-certified or DKG-not certified neurooncological centers in November 2022. Data were sourced from the directory of DKG-certified centers (https://www.oncomap.de, OnkoZert GmbH, Neu-Ulm, Germany) and the Association of University Hospitals in Germany (https://www.uniklinika.de, Association of University Hospitals in Germany e.V., Berlin, Germany, date of access November 1st 2022). A reminder email was sent after four and eight weeks. In total, the survey was open from 14th November 2022 to 24th February 2023.

The survey included questions about the location and type of the institution, the specialties involved in the neurooncology center, who conducts follow-up care for neurooncology patients, the presence of a palliative care department or coverage by other specialties, the availability of complex oncological treatment for neurooncology patients, the presence of a functioning palliative care network, the use of standardized screening tools for psycho-oncological needs or quality of life, the point at which palliative medicine physicians are involved with neurooncology patients, whether palliative treatment requires the cessation of anti-tumor therapies, the use of standardized palliative care screening for neurooncology patients, the presence of palliative care nursing outside of the palliative care unit, the availability of spiritual care concepts and chaplaincy, and the use of standardized screening for spiritual needs. The completed questionnaires were checked for plausibility by the study team. Any implausible data were resolved after consultation with the respective clinic representatives.

### Statistical analysis

Data were extracted to an Excel file (Microsoft Excel for Mac; Version 16.78, Microsoft Cooperation, Redmond, WA, USA) for analysis. Additional data were obtained directly from the directory of DKG-certified centers (https://www.oncomap.de, OnkoZert GmbH, Neu-Ulm, Germany) and the Association of University Hospitals in Germany (https://www.uniklinika.de, Association of University Hospitals in Germany e.V., Berlin, Germany, date of access: November 1st 2022).

Data were analyzed using descriptive statistics. Descriptive statistics including minimum, maximum, mean, and standard deviation (SD) were calculated for all continuous variables. Frequencies and ratios were calculated for binary variables. For statistical analyses and graphing, R studio version 2023.03.1 + 446 and Graph Pad Prism 9 for macOS (Version 9.5.0, GraphPad Software, Inc., La Jolla, CA, USA) was employed.

We also used Wilcoxon rank-sum test to compare the availability of specific specialized services between institution and certification types, as well as by geography. A p-value of < 0.05 was considered statistically significant. In addition to the survey, we collected data from the DKG website and other online sources such as hospital websites, to provide additional information about the certification process and structure of neurooncological centers.

## Results

### Geographic and institutional composition of responses

**Fig. 1:**
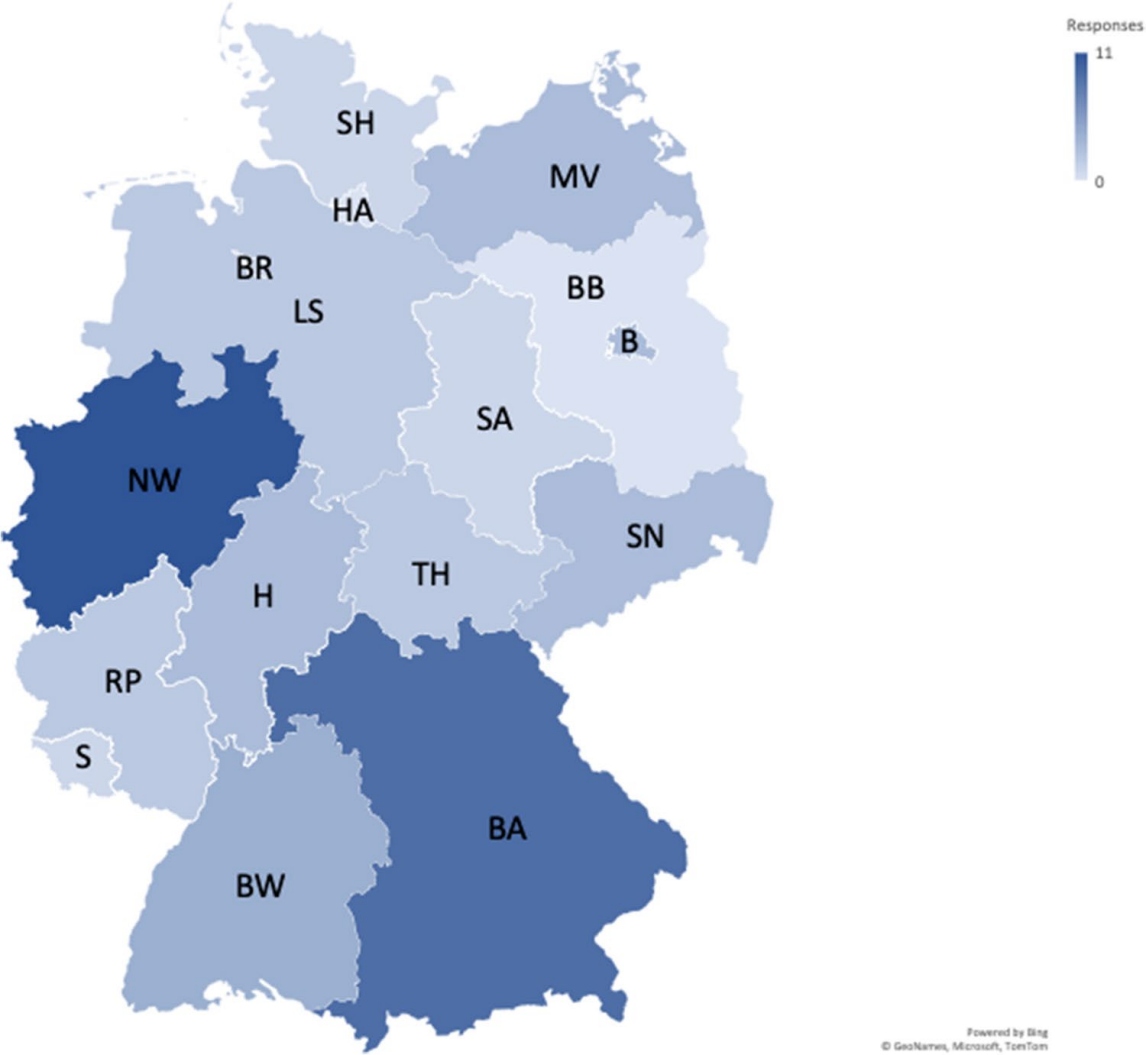
Map displaying response rates from neurooncological centers by federal state. Most responses came from North Rhine-Westphalia and Bavaria. There were no responses from Hamburg and Brandenburg (each of which have only a single DKG-certified or university-hospital-based neurooncologist center). A darker shade of blue reflects that more responses were received from that federal state. State abbreviations (with response relative to total number of centers): B = Berlin (75%); BA = Bavaria (90%); BB = Brandenburg (0%); BR = Bremen (100%); BW = Baden-Wurttemberg (57%); H = Hesse (60%); HA = Hamburg (0%); LS = Lower Saxony (100%); MV = Mecklenburg-Vorpommern (100%); NW = North Rhine-Westphalia (100%); RP = Rhineland-Palatinate (100%); S = Saarland (100%); SA = Saxony-Anhalt (50%); SH = Schleswig–Holstein (50%); SN = Saxony (100%); TH = Thuringia (100%)

A total of 46 responses were received from the 57 centers contacted, resulting in an 81% response rate. The most prominently represented federal states were the two most populous ones: North Rhine-Westphalia (n = 11) and Bavaria (n = 9). Notably, there were no responses from neurooncological centers in the federal states of Brandenburg and Hamburg, both characterized by relatively small populations. Figure 1 and Table 1 provide a comprehensive breakdown of responses by federal state.

**Table 1:** Responses by federal state in comparison with number of DKG-certified or university-hospital-based neurooncological centers in that state. Responses to the survey were obtained from institutions in all federal states except Hamburg and Brandenburg

Federal state	Responses received	Number of neurooncological centers
Baden-Wurttemberg	4	7
Bavaria	9	10
Berlin	3	4
Brandenburg	0	1
Bremen	1	1
Hamburg	0	1
Hesse	3	5
Mecklenburg-Vorpommern	3	3
Lower Saxony	2	2
North Rhine-Westphalia	11	11
Rhineland-Palatinate	2	2
Saarland	1	1
Saxony	3	3
Saxony-Anhalt	1	2
Schleswig–Holstein	1	2
Thuringia	2	2
Total	**46**	**57**

### Structure of neurooncological centers

The survey encompassed feedback from 32 university centers and 14 non-university centers.

All of the participating centers were DKG-certified. 18/46 centers were affiliated with a CCC.

**Fig. 2:**
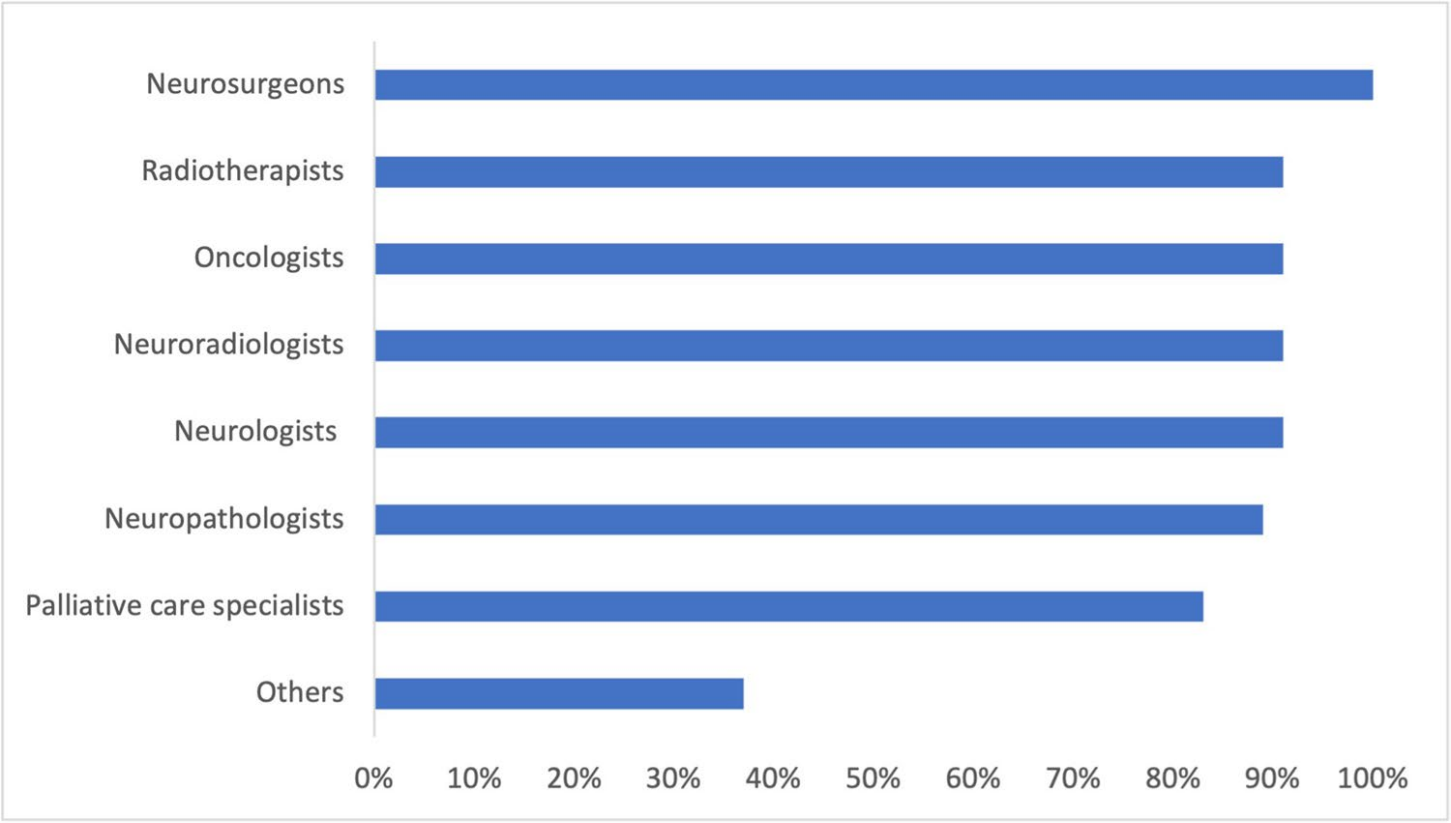
In most neurooncological centers, the core team consisted of neurosurgeons, neurologists, oncologists, radiotherapists, neuropathologists and certified palliative care specialists. (bars indicate percentage of centers)

All responders stated that a neurosurgical service formed part of their neurooncological center. In four cases, the neurooncological center was exclusively run by neurosurgeons, without direct input from other affiliated specialties. The remaining 42 neurooncological centers listed neurologists, neuroradiologists, medical oncologists and radiooncologists, neuropathologists and neuroradiologist as part of their core neurooncological team. 38/46 responders (82.6%) stated that palliative care specialists were also an essential part (Fig. 2).

According to the survey results, outpatient follow-up care for neurooncological patients involved neurosurgeons (n = 40), radiation therapists (n = 22), neurologists, and medical oncologists (n = 20 each, multiple answers were possible). In some instances, follow-up care was provided at several institutions.

### Palliative care at neurooncological centers

Dedicated palliative care departments were available at 35/46 institutions (76.1%). In addition, 31/46 responders (67.4%) stated that palliative care nurses were available to provide specialized input for patients on non-palliative wards. Palliative care physicians regularly take part in neurooncological tumor boards at only 16/46 institutions (34.8%) and patients’ palliative care needs were discussed and documented in tumor board meetings at 13/46 centers (28.3%). There was no statistically relevant difference regarding all of these aspects between organ centers and CCCs (p > 0.05, Wilcoxon rank-sum test).

44 out of 46 centers (95.7%) have well-functioning specialized palliative outpatient care networks in their regions.

### Time point of palliative care integration

There was substantial variation in the timing of involvement of palliative care specialists among the 46 neurooncological centers. The most frequently stated trigger was neurological or general deterioration (n = 22, 47.8%). Four respondents indicated that palliative treatment necessitated the cessation of all oncological therapy at their institutions. However, 14 centers (30.4%) asserted that they consistently adopt early integration, with specialized palliative care being initiated at the first diagnosis of an incurable disease in 13 centers (28.3%).

### Multidimensional assessment and treatment of complaints

Various standardized screening tools were employed to assess psychological well-being and quality of life across different centers. Palliative care burden was evaluated in 32 centers using a validated screening tool (69.6%), with 22 centers utilizing the MIDOS and 8 centers employing the IPOS. Psycho-oncological distress was measured using the distress thermometer in 37 centers (80.4%), and the Basic Documentation for Psycho-Oncology (PO-Bado) was used in 7 centers. Quality of life was assessed using the EORTC QLQ-C30 and/or EORTC QLQ-BN20 instruments in 11 centers (23.9%). A psycho-oncology service was available at all centers.

**Fig. 3:**
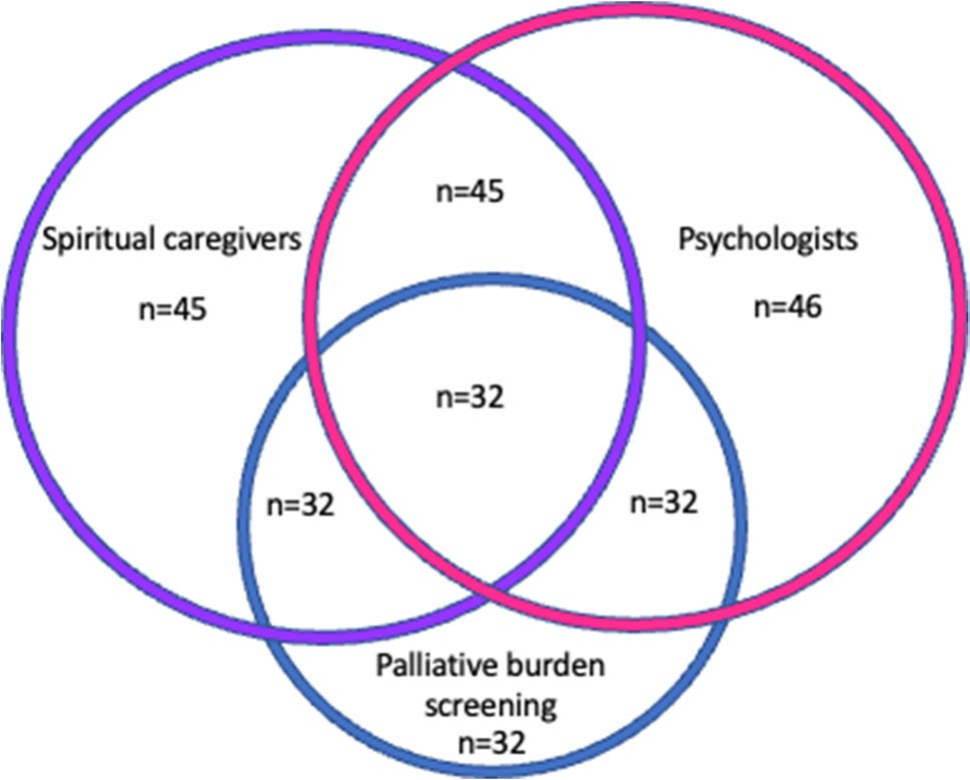
This Venn diagram reflects the concept of “total pain” in neurooncology disease and the multidisciplinary approach taken by the surveyed neurooncological centers to address its various aspects. The diagram highlights that in the majority of neurooncological centers (represented by the overlapping region), a comprehensive team is available to address the different dimensions of total pain. 32 of the surveyed centers provide a fully comprehensive approach, with standardized palliative burden screening, neurologically focused psycho-oncologists and spiritual caregivers available on a regular basis, emphasizing the widespread implementation and significance of the multidisciplinary team in addressing total pain in neurooncological disease

Spiritual caregivers were accessible in all but one institution. In the majority of centers, Christian pastors (n = 39, 84.7%) were available, and Muslim chaplains were present in at least 9 out of 46 centers (19.5%). Five out of 46 respondents (10.8%) reported the establishment of spiritual care concepts at their institutions. However, only 2 out of 46 respondents mentioned the existence of a standardized screening protocol for spiritual needs (Fig. 3).

### Advanced care planning and specialized outpatient patient care

Seventeen out of 46 representatives (36.9%) indicated that they engage in advanced care planning when managing dealing with neurooncological patients. 9/46 responders (19.6%) stated that they provided a palliative care passport for continuing care to patients as standard. Such passports contain essential information about a patient and their wishes, including regarding goals of care, advance care directives, potential treatment limitations, and faith aspects. 44/46 responders (95.6%) declared that there was a well-functioning specialized palliative out-patient care network in their region. Among these, referral to such a network was facilitated by the staff of the neurooncological center in 27 out of 46 institutions (58.7%).

## Discussion

The main results of our present study are the following:

1. Dedicated palliative care departments were present in over 75% of the responding neurooncological centers, all of which were DKG certified an/or part of university hospitals. Almost all centers have a well-functioning, specialized palliative outpatient care network in their respective regions. However, discussions and documentation of patients' palliative care needs and wishes occur in fewer than one in three tumor boards.
2. Almost half of neurooncological centers indicate that they typically engage specialized care only when patients clinically deteriorate. However, nearly 30% involve specialized palliative care at the initial diagnosis of an incurable disease.
3. Palliative care burden is routinely evaluated in 70% of centers using a validated screening tool and psycho-oncological distress was measured using the distress thermometer in 80% of centers.

### Palliative care involvement in German neurooncological centers

These survey results provide a snapshot of the palliative care provisions across leading German neurooncology centers. It demonstrates a broad spectrum of practices and approaches towards palliative care of neurooncological patients, reflecting both the inherent complexity of neurooncology and the diverse healthcare settings in which care is delivered. Considering the frequently elevated symptom burden experienced by neurooncology patients and the substantial overlap between the realms of neurooncology and palliative care, achieving comprehensive patient care hinges on interdisciplinary collaboration and the early integration of palliative care.

From our perspective, neurooncological treatment aimed at extending lifespan and palliative support complement each other. Palliative medicine extends beyond end-of-life care and its primary objective is to enhance the quality of life through multidimensional treatment of suffering within a multi-professional team. Suffering inherently encompasses physical, psychological, social, and spiritual dimensions to varying degrees [10, 11]. Therefore, the question of involvement and timing of palliative care is critical. Current guidelines strongly advocate for the early integration of (specialized) palliative care [1, 12]. Additionally, the latest ASCO guidelines recommend the inclusion of palliative care for all patients with advanced tumor disease within 8 weeks of diagnosis [13]. Approximately 75% of analyzed neurooncology centers have dedicated palliative care departments, and around 95% have a well-functioning, specialized palliative outpatient care network in their respective regions.

The initiation of palliative care demonstrates variability, with only 30% of centers involving palliative specialists soon after diagnosis. There is a notable deficiency in data regarding palliative care structures in European countries. A single-center study from the United States in 2017 highlighted limited early involvement in palliative care, with 37% of patients receiving early consultations [14]. Similarly, a literature review in the US indicated that 57% of neuro-oncological caregivers referred patients to palliative care due to symptom worsening, and 18% did so only near the end of life [15]. These findings are consistent with our survey results. Research from China and Sub-Saharan Africa also indicates a delayed engagement with palliative care specialists until late in the disease course [16, 17]. These observations suggest a need for systematic assessment and potential adjustment of the timing for palliative care integration to improve patient care.

In German neurooncological centers, the engagement of palliative care specialists within tumor board discussions is notably infrequent. Approximately a third of these centers include palliative care physicians in neurooncological tumor boards, and in less than 30% of centers are patients' palliative care needs discussed and documented during these meetings. The discussion of patients' palliative care needs and wishes, along with the introduction of a palliative care perspective, appears important. Integrating the palliative care perspective could offer crucial insights into patient needs and ensure a comprehensive care approach. Hence, we advocate that the palliative medicine perspective be incorporated into the interdisciplinary tumor board, the principal decision-making entity in Germany. Enhanced participation of palliative care physicians in these multidisciplinary dialogues could lead to more inclusive decision-making processes, as supported by recent research [18, 19]. The national divergence in practices across Germany reflects an absence of standardization, warranting further research to determine optimal timings for the involvement of palliative care in neurooncological conditions.

According to our survey, a majority of centers report the inclusion of palliative care specialists, yet the timing of this involvement exhibits considerable variation. Approximately half of the centers initiate palliative care at the onset of neurological deterioration, while under one-third incorporate these services at the diagnosis of an incurable condition. Research has demonstrated that early integration of palliative care into cancer management can significantly enhance the quality of life, alleviate symptom burden, and augment satisfaction for both patients and caregivers [20, 21]. Nevertheless, our findings indicate that only a minimal proportion of centers routinely practice early integration, highlighting an area for potential improvement.

The heterogeneity in palliative care practices, especially regarding the timing of intervention, reflects a broader diversity in approaches across the German neurooncological context. It is noteworthy that several prominent neurooncological centers, such as those in Heidelberg and Hamburg, lack certification from the DKG. This absence of DKG certification does not inherently imply a shortfall in the quality or delivery of palliative care but rather may indicate a preference for developing palliative care protocols that diverge from DKG's structured criteria. Such centers may prioritize customized strategies that better align with their distinct patient demographics and institutional goals.

### Capturing palliative care symptoms

For the successful treatment of physical, psychological, social, and spiritual complaints, their systematic recording is essential. Various German working groups have addressed the establishment of screening for psycho-oncological complaints and their systematic recording [22–26]. Moreover, systematic recording of psycho-oncological distress using a distress thermometer is considered a quality marker within the framework of the DKG certification of neurooncological centers. Accordingly, the distress thermometer is utilized in 80% of the centers, and a psycho-oncology service is available at all centers. One-fifth of the centers assess quality of life using a corresponding EORTC questionnaire. In addition to psycho-oncological screening, from a palliative medicine perspective, it is also crucial to record symptoms relevant to palliative medicine using a validated questionnaire. Palliative medicine screening is also a new quality marker as part of the DKG-based certification. Twenty-two centers utilized the MIDOS, and 8 centers employed the IPOS. The systematic documentation of palliative medical symptoms in neurooncological patients is gaining increased attention from individual working groups in Germany [27].

The survey results elucidate a notable gap in standardized protocols for spiritual care assessments and therapy, despite the recognized significance of spirituality in the context of life-threatening diseases [28]. This disparity in the provision of psychological and spiritual care prompts examination of potential provider and institutional biases, and accentuates the imperative for further investigation into the systematic standardization of spiritual care practices within neurooncological settings. Additionally, there is an apparent absence of established methodologies for documenting psychological and social complaints, with current practices limited to MIDOS, IPOS, and the Distress Thermometer [29]. As neurooncological care advances, the inclusion of structured spiritual assessments could serve as a pivotal component of a more holistic, patient-centered care paradigm.

### Continuing care and advanced care planning

The engagement of specialized inpatient or outpatient palliative care appears to be well-established in most centers. In most regions, appropriate specialized outpatient palliative care teams have been established, enabling a seamless transition. Effective oncological and palliative care also involves anticipating potential future symptoms, deteriorations, and emergency situations, and implementing precautions. This aligns with the essential aspect of advanced care planning. Encouragingly, implementation of a palliative care passport was reported at a number of centers, supporting continuity of care and respecting patient autonomy. With regard to advanced care planning, only about one third of the centers reported that this is a routine practice. These findings are in accordance with previous studies. A meta-analysis estimated that fewer than 40% of primary brain tumor patients engaged in end-of-life discussions covering decisions about treatment preferences, health care proxy, palliative care consultation, hospice, and resuscitation wishes before their death [30]. The percentage of patients with a complete advance directive (AD), documenting their wishes and/or appointing a substitute decision-maker, ranged from 0 to 76%. These rates varied significantly among different countries, including within Europe. In a recent Norwegian paper, 78% of brain tumor patients completed palliative care decisions [31]. Previous studies have emphasized the benefits of advanced care planning in improving end-of-life care and reducing hospital admissions and recent recommendation advise early palliative care interventions and advance care planning as part of each disease phase, proactively addressing different patient and caregiver need [32].

## Study limitations

This study offers valuable insights into palliative care practices in neurooncology within Germany, yet it is important to acknowledge certain limitations that influence the interpretation of its findings. The response rate, while high at 81%, suggests the potential for non-response bias, which might impact the representativeness of the results. Additionally, the absence of responses from two German states could mean that the study does not entirely encapsulate the national scope of palliative care practices in neurooncology centers.

One of the study’s constraints arises from its reliance on self-reported data from participating centers. Although self-reporting is an effective method for gathering a wide-ranging overview of practices, it carries inherent susceptibilities to subjective biases. These may manifest in the form of response bias, where respondents might inadvertently present their practices in a more favorable manner. The lack of external verification of this data further adds a layer of complexity to the study, highlighting the need for corroborative research methods in future studies to strengthen the findings.

Focusing predominantly on university and DKG-certified neurooncological centers provides a comprehensive look at established institutions, yet this approach may overlook the varied practices at non-university and non-certified centers. These centers could potentially harbor unique and innovative palliative care approaches that could enrich the overall understanding of palliative care in neurooncology.

The study's exploration primarily centers on structural aspects of palliative care, without an extensive examination of the quality of care or comparative effectiveness of different practices. Additionally, the case loads of each institution and the extent of palliative care involvement in neurooncological cases were not assessed, which could have added valuable depth to the study. Future studies could further explore this aspect, as well as investigate patient and caregiver perspectives to provide a more comprehensive evaluation.

This study evaluated the palliative care for neuro-oncological patients in general. Further exploration of palliative care aspects in specific diagnoses may warrant valuable insights into neuro-oncology practices and help identify areas for improvement. A recent study specifically assessed availability of palliative care for inpatients with malignant gliomas in Germany and found that only 10% of these patients received specialized palliative care [33]. Additional research is necessary to assess palliative care aspects in other diagnoses, such as parenchymal or leptomeningeal metastatic disease.

The generalizability of the findings is also a point to consider. Given that the study is situated within the specific context of the German healthcare system, with its distinct structure, funding mechanisms, and policy frameworks, extrapolation of these findings to different healthcare systems requires careful consideration. While this does not diminish the study's contributions, it does underscore the necessity of interpreting the results as reflective of the German healthcare environment, rather than being universally representative.

In summary, this study makes a significant contribution to the field of neurooncological palliative care, particularly within the German context. Its findings lay the groundwork for future research, which should ideally encompass a wider range of healthcare institutions and employ varied research methodologies to provide a more comprehensive and nuanced understanding of palliative care practices in neurooncology.

## Conclusion

Our findings illuminate the diverse landscape of palliative care provision in German neurooncology centers, and will prove valuable for neurooncology centers, policymakers, and other stakeholders working to improve patient care. While there are clear efforts to integrate palliative care into the overall management of neurooncological patients, the timing and depth of this integration vary. There is a need to strengthen the early integration of palliative care, enhance participation of palliative care specialists in multidisciplinary tumor boards, and increase focus on advanced care planning. The standardization of palliative care burden, psychological and spiritual screening tools could also enhance holistic care delivery.

As our understanding of the complexities of neurooncology continues to evolve, so too should our approach to palliative care. The goal must always be to ensure that patients receive compassionate, comprehensive, and personalized care that maximizes quality of life and honors their wishes and autonomy. These findings offer a valuable foundation for further discussion and for the development of national guidelines and protocols for the provision of palliative care in neurooncology.

### Supplementary Information

Below is the link to the electronic supplementary material.

Supplementary file1 (DOCX 15 KB)

Supplementary file2 (DOCX 32 KB)
