# Supplementary material for: Benchmarking palliative care practices in neurooncology: a german perspective
Source: J Neurooncol. 2024 May 2;168(2):333–43. doi: 10.1007/s11060-024-04674-7 (PMC11147867; doi:10.1007/s11060-024-04674-7)
Supplement: Supplementary file 1 — Supplementary file1 (DOCX 15 KB) [file 11060_2024_4674_MOESM1_ESM.docx]

Members of the German Consortium for Excellence in Neurooncology and Palliative Care (GCE-NPC) – to be listed as collaborators in PubMed

| Name | Institution | Contact |
| --- | --- | --- |
| Martin Misch | Department of Neurosurgery, Charité, University Hospital Berlin, Germany | martin.misch@charite.de |
| Ulrich Herrlinger | Division of Clinical Neurooncology, Dep. of Neurology and Center of Integrated Oncology (CIO) Bonn, Germany | ulrich.herrlinger@ukbonn.de |
| Vesna Malinova | Department of Neurosurgery, University Hospital Göttingen, Germany | vesna.malinova@med.uni-goettingen.de |
| Marcia Machein | Department of Neurosurgery, University Hospital Freiburg, Germany | marcia.machein@uniklinik-freiburg.de |
| Peter Hau | [Department](mailto:peter.hau@ukr.de) of Neurology, University Hospital Regensburg, Germany | peter.hau@ukr.de |
| Oliver Grauer | Department of Neurology, University Hospital Münster, Germany | oliver.grauer@ukmuenster.de |
| Martin Glas | Department of Clinical Neurooncology, Department of Neurology, University Hospital Essen, Germany | Martin.Glas@uk-essen.de |
| [Almuth F. Kessler](mailto:kessler_a1@ukw.de) | Department of Neurosurgery, University Hospital Würzburg, Germany | [kessler_a1@ukw.de](mailto:kessler_a1@ukw.de) |
| [Naureen Keric](mailto:naureen.keric@unimedizin-mainz.de) | Department of Neurosurgery, University Hospital Mainz, Germany | naureen.keric@unimedizin-mainz.de |
| Hannes Egermann | Department of Neurosurgery, Barmherzige Brüder Hospital, Regensburg, Germany | hannes.egermann@barmherzige-regensburg.de |
| Marco Stein | Neurooncological Centre, University Hospital Gießen/Marburg, Campus Gießen, Germany | marco.stein@neuro.med.uni-giessen.de |
| Jens Weigel | Department of Neurosurgery, Paracelsus Private Medical School, Nuremburg, Germany | jens.weigel@klinikum-nuernberg.de |
| Marcus Reinges | Department of Neurosurgery, Hospital Bremen-Mitte, Bremen, Germany | Marcus.Reinges@gesundheitnord.de |
| [Andreas Jödicke](mailto:andreas.joedicke@vivantes.de) | Neurooncological Center, CCBN, Department of Neurosurgery, Vivantes Hosiptal Berlin, Germany | andreas.joedicke@vivantes.de |
| Klaus-Peter Stein | Department of Neurosurgery, University Hospital Magdeburg, Germany | peter.stein@med.ovgu.de |
| Marcus Mehlitz | Department of Neurosurgery, Barmherzige Brüder Hospital, Trier, Germany | m.mehlitz@bbtgruppe.de |
| [Sven-Axel May](mailto:s-a.may@skc.de) | Department of Neurosurgery, Chemnitz Hospital, Germany | s-a.may@skc.de |
| Niklas Thon | Department of Neurosurgery, University Hospital of the Lukas-Maximilian-University Munich, Germany | niklas.thon@med.uni-muenchen.de |
| [Rebecca Kassubek,](mailto:rebecca.kassubek@uni-ulm.de) | Department of Neurology, University Hospital Ulm, Germany | rebecca.kassubek@uni-ulm.de |
| Ulrich Knappe | Neurooncological Center MiNOZ, Johannes Wesling Medical Center Minden, UK RUB, Minden, Germany | neurochirurgie@muehlenkreiskliniken.de |
| Ali Alomari | Neurooncological Center MiNOZ, Johannes Wesling Medical Center Minden, UK RUB, Minden, Germany | neurochirurgie@muehlenkreiskliniken.de |
| [Florian H. Ebner](mailto:florian.ebner@krupp-krankenhaus.de) | Department of Neurosurgery, Alfried Krupp Hospital, Essen Rüttenscheid, Germany | florian.ebner@krupp-krankenhaus.de |
| Mirjam Renovanz | Neurooncological Center, University Hospital Tübingen, Germany | mirjam.renovanz@med.uni-tuebingen.de |
| Elisabeth Bumes | Elisabeth Bumes, Wilhelm Sander-NeuroOncology Unit, Regensburg University Hospital, Regensburg, Germany | elisabeth.bumes@ukr.de |
| Clemens Seidel | Department of Radiooncology, University Hospital Leipzig, Germany | clemens.seidel@medizin.uni-leipzig.de |
| [Hans Clusmann](mailto:hclusmann@ukaachen.de) | Department of Neurosurgery, University Hospital RWTH Aachen, Germany | hclusmann@ukaachen.de |
| Thomas M. Freiman | Department of Neurosurgery, University Hospital Rostock, Germany | thomas.freiman@med.uni-rostock.de |
| Yu-Mi Ryang | Department of Neurosurgery, Helios Hospital Berlin Buch, Berlin, Germany | Yu-Mi.Ryang@helios-gesundheit.de |
| [Julia Gerhardt](mailto:Yu-Mi.Ryang@helios-gesundheit.de) | Department of Neurosurgery, Helios Hospital Berlin Buch, Berlin, Germany | julia.gerhardt@helios-gesundheit.de |
| [Michael Stoffel](mailto:michael.stoffel@helios-gesundheit.de) | Department of Neurosurgery, Helios Hospital Krefeld, Germany | michael.stoffel@helios-gesundheit.de |
| [Ina Lange](mailto:ina.lange@med.uni-greifswald.de) | Neurooncological Center Greifswald, University Hospital Greifswald, Germany | ina.lange@med.uni-greifswald.de |
| Volker Tronnier | Department of Neurosurgery, University Hospital Schleswig-Holstein, Campus Lübeck, Germany | volker.tronnier@neurochirurgie.uni.luebeck.de |
| Walter Schulz-Schaeffer | Institute of Neuropathology, University Hospital Homburg/Saar, Germany | Walter.Schulz-Schaeffer@uks.eu |
