# Supplementary material for: Benchmarking palliative care practices in neurooncology: a german perspective
Source: J Neurooncol. 2024 May 2;168(2):333–43. doi: 10.1007/s11060-024-04674-7 (PMC11147867; doi:10.1007/s11060-024-04674-7)
Supplement: Supplementary file 2 — Supplementary file2 (DOCX 32 KB) [file 11060_2024_4674_MOESM2_ESM.docx]

**Umfrage zum Thema Palliativmedizin in der Neuroonkologie**

Sehr geehrte Damen und Herren,

wir möchten die palliativmedizinische Versorgung neuroonkologischer PatientInnen in Deutschland evaluieren. Aus diesem Grund bitten wir Sie um die Beantwortung von 26 kurzen Fragen zur Behandlung von Patienten in Ihrem neuroonkologischen Zentrum. Sie können uns freiwillig Ihren Namen und Ihre Institution nennen. In diesem Fall werden wir Sie als auf PubMed-gelistete Mitwirkende (Collaborators) in einer resultierenden Publikation listen. Die Auswertung wird aber unabhängig von diesen Angaben auf Bundeslandebene ohne explizite Nennung von Institutionen oder Individuen erfolgen.

Wir bedanken uns für Ihre Teilnahme!

Für Rückfragen stehen wir gern unter der E-Mail-Adresse anna.lawsonmclean@med.uni-jena.de zur Verfügung.

Mit freundlichen Grüßen

Marcel Kamp, Aaron McLean & Anna McLean

vom Zentrum für Neuroonkologie der Klinik und Poliklinik für Neurochirurgie am Universitätsklinikum Jena

1. **In welchem Bundesland befindet sich Ihre Einrichtung?**
2. Baden-Württemberg
3. Bayern
4. Berlin
5. Brandenburg
6. Bremen
7. Hamburg
8. Hessen
9. Mecklenburg-Vorpommern
10. Niedersachsen
11. Nordrhein-Westfalen
12. Rheinland-Pfalz
13. Saarland
14. Sachsen
15. Sachsen-Anhalt
16. Schleswig-Holstein
17. Thüringen
18. **Handelt es sich um ein Universitätsklinikum oder eine nichtuniversitäre Einrichtung?**
19. Universitätsklinikum
20. Nicht-universitäre Einrichtung
21. **Welchen Zertifizierungsstatus hat Ihre Einrichtung?**
22. Comprehensive Cancer Center
23. Organzentrum Neuroonkologie
24. Nicht zertifiziert
25. **Welche Fachgebiete sind in Ihrem neuroonkologischen Zentrum beteiligt? (Mehrfachnennung möglich)**
26. Neurochirurgie
27. Neurologie
28. Neuropathologie
29. Neuroradiologie
30. Onkologie
31. Palliativmedizin
32. Strahlentherapie
33. Weitere
34. **Wer führt das ambulante Follow-up für neuroonkologische PatientInnen durch? (Mehrfachnennung möglich)**
35. Neurochirurgie
36. Neurologie
37. Onkologie
38. Strahlentherapie
39. Außer Haus
40. **Ist die Palliativmedizin in Ihrem Hause eine eigenständige Abteilung oder wird durch andere Fachdisziplinen (z.B. Onkologie, Anästhesie) mitabgedeckt**?
41. Keine Palliativmedizin im Haus vorhanden
42. Palliativmedizin wird über andere Fachdisziplinen abgedeckt
43. Eigenständige palliativmedizinische Klinik / Abteilung
44. **Ist in Ihrem Zentrum eine onkologische Komplexbehandlung für neuroonkologische PatientInnen stationär möglich?**
45. Ja
46. Nein
47. **Gibt es in Ihrem Einzugsbereich ein funktionierendes SAPV-Netzwerk?**
48. Ja
49. Ja, allerdings mit Lücken
50. Nein
51. **Durch wen wird in der Regel eine spezialisierte ambulante palliativmedizinische Versorgung (SAPV) verordnet?**
52. Durch das neuroonkologische Zentrum
53. Durch den Hausarzt bzw. die Hausärztin
54. Durch andere
55. **Wird Palliativbedarf regelhaft in Ihrer neuroonkologischen Tumorkonferenz besprochen und dokumentiert?**
56. Ja
57. Nein
58. **Nimmt ein Palliativmediziner bzw. eine Palliativmedizinerin regelmäßig an Ihrer neuroonkologischen Tumorkonferenz teil?**
59. Ja
60. Nein
61. **Wird standardmäßig eine sozialrechtliche Beratung durch den Sozialdienst bei Erstdiagnose durchgeführt?**
62. Ja
63. Nein
64. **Gibt es in Ihrer Einrichtung psycho-onkologisch spezialisierte PsychologInnen?**
65. Ja
66. Nein
67. **Gibt es ein standardisiertes Screening für psycho-onkologischen Bedarf bzw. ein Screening für Lebensqualität? (Mehrfachnennung möglich)**
68. Basic Documentation for Psycho-Oncology (PO-Bado)
69. Distress-Thermometer
70. EORTC QLQ-C30 +/- EORTC QLQ-BN20
71. Hospital Anxiety and Depression Scale (HADS)
72. Anderes Instrument
73. Kein standardisiertes Screening
74. **Zu welchem Zeitpunkt werden PalliativmedizinerInnen bei PatientInnen mit malignen neuroonkologischen Erkrankungen involviert? (Mehrfachnennung möglich)**
75. Kein Zeitpunkt wurde für die Involvierung eines Palliativmediziners bzw. einer Palliativmedizinerin definiert
76. Bei Erstdiagnose einer nicht heilbaren und / oder lebensbedrohlichen Tumorerkrankung
77. Zu einem definierten Zeitpunkt im Krankheitsverlauf (z.B. 1. Tumorrezidiv)
78. Bei neurologischer oder AZ-Verschlechterung zu einem definierten Zeitpunkt
79. Nach Absetzen aller gegen den Tumor gerichteten Therapien (z.B. Chemo-, Immun-, Strahlentherapien)
80. Auf Patientenanfrage
81. **Erfordert eine palliativmedizinische Behandlung von PatientInnen mit malignen neuroonkologischen Erkrankungen in Ihrem Hause ein Absetzen jeglicher anti-tumoröser Therapien?**
82. Ja
83. Nein
84. **Gibt es ein standardisiertes palliativmedizinsches Screening für alle neuro-onkologischen PatientInnen (mit malignen Erkrankungen)?**
85. Ja
86. Nein
87. **Falls ja, welches palliativmedizinische Screening-Instrument wird verwendet?**
88. Edmonton Symptom Assessment System (ESAS)
89. Minimal Documentation System (MIDOS)
90. Palliativmedizinisches Basisassessment (PBA)
91. Anderes Instrument
92. **Ist ein standardmäßiges Vorgehen im Falle eines auffälligen palliativmedizinischen Screenings hinterlegt?**
93. Ja
94. Nein
95. **Gibt es in Ihrem Zentrum außerhalb der Palliativstation eine palliativmedizinische Fachpflege?**
96. Ja
97. Nein
98. **Gibt es in Ihrem Zentrum Spiritual Care Konzepte? Falls ja, welche?**

{Antwort als Freitext}

1. **Gibt es in Ihrer Einrichtung eine/n täglich verfügbare/n Seelsorger/in?**
2. Ja
3. Nein
4. **Wenn ja, für welche Konfessionen bzw. Religionen? (Mehrfachnennung möglich)**

{Antwort als Freitext}

1. **Gibt es ein standardisiertes Screening für Seelsorge oder spirituellen Bedarf?**
2. Ja
3. Nein
4. **Falls ja, welches Screening-Instrument verwenden Sie?**

{Antwort als Freitext}

1. **Sind folgende (palliativ-medizinische) Konzepte in Ihrem neuroonkologischen Zentrum routinemäßig etabliert? (Mehrfachnennung möglich)**
2. Advanced care planning
3. Early integration
4. Palliativausweis mit Verfügung über Krankenhauseinweisung
5. Shared decision making
6. Total pain bzw. total suffering
7. Keines der o.g. Konzepte
8. **Freiwillig: Kompletter Name, Einrichtung und E-Mail-Adresse (wenn Sie als PubMed-gelistete/r Mitwirkende/r (Collaborator) an dem aus dieser Umfrage resultierenden Artikel aufgeführt werden möchten)?**

Nachname:

Vorname:

Institution:

Abteilung:

E-Mail-Adresse:

**Survey on palliative care in neuro-oncology – English translation**

Dear Sir or Madam,

We would like to evaluate the current state of palliative care of neuro-oncology patients in Germany. For this reason, we would like to ask you to answer 26 short questions about the treatment of patients in your neuro-oncology center. You can voluntarily state your name and institution at the end of the survey. In this case, we will list you as a PubMed-listed collaborator in a resulting publication. However, the evaluation will be carried out independently of this information at federal state level without explicitly naming institutions or individuals.

Thank you for your participation!

If you have any questions, please do not hesitate to contact us at anna.lawsonmclean@med.uni-jena.de.

Yours sincerely

Marcel Kamp, Aaron McLean & Anna McLean

Neurooncological Center, Department of Neurosurgery, Jena University Hospital

1. In which federal state is your institution located?

a) Baden-Wurttemberg

b) Bavaria

c) Berlin

d) Brandenburg

e) Bremen

f) Hamburg

g) Hesse

h) Mecklenburg-Western Pomerania

i) Lower Saxony

j North Rhine-Westphalia

k) Rhineland-Palatinate

l) Saarland

m) Saxony

n) Saxony-Anhalt

o) Schleswig-Holstein

p) Thuringia

2. Is it a university hospital or a non-university institution?

a) University hospital

b) Non-university institution

3. What certification status does your institution have?

a) Comprehensive Cancer Center

b) Organ Center Neurooncology

c) Not certified

4) Which specialties are involved in your neuro-oncology center? (multiple answers possible)

a) Neurosurgery

b) Neurology

c) Neuropathology

d) Neuroradiology

e) Oncology

f) Palliative medicine

g) Radiotherapy

h) Others

5. Who carries out the outpatient follow-up for neuro-oncology patients? (multiple answers possible)

a) Neurosurgery

b) Neurology

c) Oncology

d) Radiotherapy

e) Off-site

6) Is palliative medicine an independent department in your hospital or is it also covered by other specialist disciplines (e.g. oncology, anesthesia)?

a) No palliative care available

b) Palliative care is covered by other specialist disciplines

c) Specialized palliative care department

7. Is a standardized oncological treatment for neuro-oncological patients (“onkologische Komplexversorgung”) available for inpatients at your institution?

a) Yes

b) No

8. Is there a functioning specialized outpatient palliative care network available in your catchment area?

a) Yes

b) Yes, but with gaps

c) No

9) Who usually prescribes specialized outpatient palliative care?

a) Neuro-oncology center

b) General practitioners / family doctors

c) Others

10) Is the need for palliative care regularly discussed and documented in your neuro-oncological tumor conference?

a) Yes

b) No

11. Does a palliative care physician regularly take part in your neuro-oncological tumor conference?

a) Yes

b) No

12. Is it standard practice for the social services department to provide socio-legal advice on initial diagnosis?

a) Yes

b) No

13. Are there psycho-oncologically specialized psychologists in your facility?

a) Yes

b) No

14. Is there standardized screening for psycho-oncological needs or a screening for quality of life? (multiple answers possible)

a) Basic Documentation for Psycho-Oncology (PO-Bado)

b) Distress Thermometer

c) EORTC QLQ-C30 +/- EORTC QLQ-BN20

d) Hospital Anxiety and Depression Scale (HADS)

e) Other screening instrument

f) No standardized screening

15. At what point are palliative care physicians involved in patients with malignant neuro-oncological diseases? (multiple answers possible)

a) No point in time was defined for the involvement of a palliative care physician

b) At the initial diagnosis of an incurable and/or life-threatening tumor disease

c) At a defined point in the course of the disease (e.g. 1st tumor recurrence)

d) In the event of neurological or general deterioration at a defined point in time

e) After discontinuation of all therapies directed against the tumor (e.g. chemotherapy, immunotherapy, radiotherapy)

f) On patient request

16) Does palliative medical treatment of patients with malignant neuro-oncological diseases in your hospital require discontinuation of all anti-tumor therapies?

a) Yes

b) No

17. Is there a standardized palliative care screening for all neuro-oncological patients (with malignant diseases)?

a) Yes

b) No

21 Does your center have spiritual care concepts? If yes, which ones?

{answer as free text}

22. Is there a chaplain available daily at your center?

a) Yes

b) No

23. If yes, for which denominations or religions? (multiple answers possible)

{answer as free text}

24. Is there a standardized screening for pastoral care or spiritual needs?

a) Yes

b) No

25) If yes, which screening tool do you use?

{answer as free text}

26. Are the following (palliative care) concepts routinely established in your neuro-oncology center? (multiple answers possible)

a) Advanced care planning

b) Early integration

c) Palliative care card with decision on hospitalization

d) Shared decision making

e) Total pain or total suffering

f) None of the above concepts

27 Voluntary: Full name, institution and e-mail address (if you wish to be listed as a PubMed-listed collaborator on the article resulting from this survey)?

Last Name:

First name:

Institution:

Department:

E-mail address:
